# Gastric Cancer in History: A Perspective Interdisciplinary Study

**DOI:** 10.3390/cancers12020264

**Published:** 2020-01-22

**Authors:** Skender Topi, Luigi Santacroce, Lucrezia Bottalico, Andrea Ballini, Alessio Danilo Inchingolo, Gianna Dipalma, Ioannis Alexandros Charitos, Francesco Inchingolo

**Affiliations:** 1 Department of Clinical Disciplines, School of Technical Medical Sciences, University of Elbasan “A. Xhuvani”, 3001 Elbasan, Albania; skender.topi@uniel.edu.al; 2 Surgery, Regional Hospital “X. Kongoli”, 3001 Elbasan, Albania; 3 Ionian Department (DJSGEM), Microbiology and Virology Lab., University of Bari “A. Moro”, 70124 Bari, Italy; 4 Polypheno Academic Spin Off, University of Bari “A. Moro”, 70124 Bari, Italy; bottalico.lu@gmail.com; 5 Department of Biosciences, Biotechnologies and Biopharmaceutics, University of Bari “Aldo Moro”, 70124 Bari, Italy; andrea.ballini@uniba.it; 6 Department of Precision Medicine, University of Campania “Luigi Vanvitelli”, S. Andrea delle Dame—Via L. De Crecchio, 7—80138 Naples, Italy; 7 Department of Interdisciplinary Medicine, University of Bari, General Hospital, Bari, Italy; aleinki10@hotmail.it (A.D.I.); info@francescoinchingolo.it (G.D.); francesco.inchingolo@uniba.it (F.I.); 8 Poisoning National Center, Emergency and Urgency Service, Riuniti University Hospital of Foggia, 71122 Foggia, Italy; alexanestesia@hotmail.com

**Keywords:** gastric cancer, *Helicobacter pylori*, gastritis, atrophic gastritis, peptic ulcer disease (PUD), gastric surgery, epidemiology, history of medicine, famous patients, personalized medicine

## Abstract

Background: Gastric adenocarcinoma is the fourth most common type of cancer and the second leading cause of cancer death in the world. Despite abundant traces of an ancient history, the comprehension of its pathogenic mechanisms is rather recent and continuously updated. Methods: We investigated about how the ancient civilizations tried to understand the exactly physiopathology of gastric cancer, from the time when they could not examine deeply the histological and pathophysiologic aspects of the disease, but they just based their knowledge on a visual analysis of the signs and consequences of such disease. We examined the historical evolving knowledge of the disease along the centuries on the gastroenterological, pharmacological, and surgical fields, defining how gastric cancer became an increasingly curable disease. Results: Cancer was known in the ancient world. Ancient people did not know exactly the causes but the climatic, hygienic, and food conditions were the first to be considered over time, also taking into consideration supernatural negative influences. During the Renaissance, a tumultuous time of scientific discoveries started, thanks to an increasing number of autopsies made on cadavers and to the progressions in visual analysis of the stomach mucosa throughout endoscopy. From the first gastric surgery in 1879, many steps forward have been made and, today, gastric cancer is regarded as a more curable disease; one important discovery in this field has been the revelation of the role of *Helicobacter pylori* in the peptic ulcer disease (PUD) and in some forms of gastric lymphoma. Conclusions: Gastric cancer has the fourth highest incidence of various cancers worldwide and is ranked second as a cause of cancer-related death. It exists from the antiquity and a lot of hypotheses have been developed about its etiology during the centuries, influencing its therapy. During the 20th century, thanks to the scientific and technological progresses the causes of the cancer have been discovered and the role of the bacterium *Helicobacter pylori* has been demonstrated, and new perspective research are currently trying to investigate the role of other microorganisms in gastric physiopathology, as well as its possible modulation by probiotics.

## 1. Introduction

Gastric cancer is a well-known, life threatening condition affecting many people worldwide. Today, gastric cancer represents one of the leading causes of death worldwide, but very little is known about the antiquity, epidemiology and evolution of cancer in past human populations [1]. Currently, the incidence is decreasing thanks to early diagnosis, reduced incidence of *Helicobacter pylori* infection, diffusion of healthy lifestyles (including smoke cessation and good nutrition), but some geographical differences still remain in its incidence and mortality rate.

However, during the times gastric cancer accounted for a large number of deaths, mainly because of the lack of knowledge about its causes and of the absence of effective therapies. In fact, the first official surgical intervention for gastric cancer was performed in the late 19th century thanks to Prof, Theodor Billroth.

If the therapy of gastric cancer is recent, its history is very long, as reported in some documents from ancient civilizations. Starting from our own experience as surgeons and anesthesiologists, oral health professionals, pathologists, microbiologist and laboratory clinicians, we have focused the main steps of the evolution of the knowledge about gastric cancer, its etiology, and treatments through time.

For this purpose, selected articles were identified through multiple formal search methods including hand searching of key journals; electronic searching of main databases including the use of free-text, index terms and named author. Electronic searches of the following databases were conducted: Web of Knowledge, Web of Science, Google Scholar, Elsevier’s (EMBASE.com), PubMed, Medline, National Library of Greece (Stavros Niarchos Foundation, Athens, Greece), and the Library of the school of Health Sciences of the National and Kapodistrian University of Athens. Search terms such as “History of gastric cancer”, “Gastric surgery history”, and “Helicobacter pylori discovery” were used in combination to further focus and limit the selection to historical articles, including papers published in peer-reviewed English-language journals and books from as early as 1937 through to 2019.

## 2. The Paleo-Oncology Evidence and the Earlier Studies

Ancient medical texts which describe pathological conditions, identified as cancer, were written both by the Ancient Egyptians and Greeks. One of the earliest known description of cancer first appeared in different papyri from Egypt, such as the Kahun (or Fayoum) papyrus (1950 BC), which consists of three sections, with one relating to human medicine, the “*Edwin Smith*” papyrus which was written approximately in 1600 BC (but supposed to be a copy of a document dating back to 3000 BC) and, finally, the “Ebers Papyrus” (1600 BC), which is considered among the most important medical papyri of ancient Egypt; contains the first reference to possible cancers of the skin, uterus, stomach, and rectum. Those ancient texts treated cancers with hot blades, knives, salts, and arsenic paste [2,3,4,5]. In his tractate on “Ancient Egyptian Medicine”, John F. Nunn draws on his own clinical experience as a physician and an Egyptologist to reassess the evidence: he translated and reviewed the original Egyptian medical papyri, reconsidering other sources of information, including mummies, skeletons, tomb paintings, statues, and coffins. He described in his work the criteria by which the Egyptian doctors made their diagnoses of several diseases, including gastrointestinal ones [5]. During the period of the tenth dynasty of the pharaohs (2125 BC), Irynakhty was the court physician, who conducted extensive studies in the field of gastroenterology and proctology [5].

It is very difficult for archeologists and physicians to diagnose cancer in human skeletons and mummified individuals because differentiation from other lytic pathologies is not always possible when based only on morphological appearance but also because the skeletal collections are often confined to skulls or selected pathological bones. The distribution and density of metastatic lesions depend on the primary cancer: malignant tumors which cause osteolytic lesions are, for example, thyroid, kidneys, adrenal glands, uterus, and gastrointestinal tract tumors. To date, only around 200 cases have been reported. A case of multiple metastatic lesions due to cancer was found in the archaeological site of Amara West in Northern Sudan (1200 BC): a male, young-adult individual displaying multiple osteolytic lesions on his bones. After a radiographic, microscopic, and scanning electron microscopic imaging of the lesions, and a consideration of differential diagnoses, a diagnosis of metastatic carcinoma secondary to an unknown soft tissue cancer was suggested. This is the earliest complete example in the world of a human who suffered metastatic cancer to date [6,7].

One of the first Greek physicians and physiologists of the pre-Hippocratic medicine was Alcmaeon of Croton (Αλκμαίων ὁ Κροτωνιάτης), in the 6th century BC. Some studies about his work as a physician clearly demonstrate his knowledge about some gastrointestinal mechanisms and digestive functions that he tried to study throughout the animal’s vivisection [8].

Afterwards, one reference about a possible gastric cancer was reported also by Hippocrates of Kos (Ἱπποκράτης ὁ Κῷος, 460-377 BC) in the 4th BC. He believed that this pathology attacked the human body from the outside, penetrating through the skin and infiltrating tissues and internal organs. According to legend, he was the first to use the words “καρκίνος” (karkìnos = cancer), and “καρκίνωμα” (karkìnoma = carcinoma) because of the finger-shaped projections that spread out of the tumor resemble the shape of the crab’s fingers. Until then, without a sectional anatomy, all the diseases were considered to be caused by the absorption of black bile from the bowel into the blood, but also by the poor quality of environmental air and were cured by purging, enemas, and blood-lettings [9]. Indeed, in his medical work “Περί αέρων, τόπων, υδάτων” (About Air, Earth and Water), he described the hydrotherapy efficacy [10].

Very interested in gastrointestinal diseases and their possible remedies were the Hellenistic Alexandria of Egypt medical school’s scholars (288–300 BC) and was founded by Herophilus of Chalcedon (Ἡρόφιλος ὁ Χαλκηδών, 330–260 BC), an Alexandrian physician, received his medical training under Praxagoras of Kos (Πραξαγόρας ὁ Κῷος, 4th–3th C. BC) a famous physician and anatomist who taught at the Hippocratic medical school on the island of Kos. With his contemporary colleague, Erasistratus of Ceos (Ἐρασίστρατος, 304–250 BC), he practiced a large number of dissections on animal’s bodies to study anatomy; however, they conducted an exceptional activity: the first ever scientific human dissections on cadavers. Human dissections then were not allowed again for 1800 years. It seems that only these two physicians ever performed human dissections until the Renaissance, around 1530 AC, Herophilus’ discoveries on human body were basilar for human medicine, so is called the Father of Anatomy [11]. Herophilus and Erasistratus were two of an important group of great Greek physicians of the Alexandria medical school who dedicated great deal of time studying human medicine and tried to achieve a large knowledge about several human diseases and their possible treatments. Subsequently, at the Roman ages the Greek physician Asclepiades of Bithynia (Ἀσκληπιάδης της Βιθυνίας, 124–40 BC) studied medicine in Athens and in Alexandria of Egypt, practiced in Rome. He was the founder of the “Methodic school” (Μεθοδική Σχολή), a very important center for medical studies. He used to treat gastrointestinal diseases with mixed drinks based on wine and plant’s extracts [12]. Indeed, in the “De Materia Medica” by Dioscorides Pedanius (Διοσκουρίδης Πεδάνιος, 40–90 AC), the Greek doctor who is considered the father of pharmacology, influenced by the theories of Asclepiades, he talked about several possible treatments of gastrointestinal symptoms, using a drink based on wine and sometimes honey, mixed with different plant’s extracts. Some wine-based preparations with their specific names and indications were: avronitis (*αβροτονίτης*) with *Absinthium ponticum* or *Artemisia afra* or *Artemisia abrotanum*, thrimbitis (*θυμβρίτης*) with *Satureja thymbra*, thimitis (*θυμίτης*) with *Thymus vulgaris* or *Coridothymus capitatus*), origanitis (*οριγανίτης*) with *Οriganum vulgare*, calaminthitis (*καλαμινθίτης*) with *Thymus calamintha*, gliconitis (*γληχωνίτης*) with *Mentha pulegium*, for dyspepsia and abdominal pains, apitis (*απίτης*) with *Pyrus communis*, apsinthitis (*αψινθίτης*) with *Artemisia absinthium*, mirtitis (*μυρτίτης*) with *Myrtus communis* or *Valeriana dioscoridis* οr *V. italica lam.* or *V. officinalis*, nectaritis (*νεκταρίτης*) with *Inula helenium*, roitis (*ροΐτης*) with *Punica granatum* or *Scilla maritime* were considerate a good remedy for stomach diseases [12,13].

The theories of Hippocrates reached the Roman age, where were shared by the Greek physician Galen of Pergamon (Γαληνός ὁ Περγαμηνός, 130–200 AC), who first used the term “όγκος” (tumor) to describe cancer. He also used the Greek prefix “onco-” (*όγκο*-, which is the Greek term for “swelling”) and the suffix “-oma” (*-ομα*) to describe tumors in general, reserving the Hippocratic term “carcinoma” for malignant ones. These ancient terms are currently used in the actual oncology [14].

In the Early Middle Ages, a large and complete classification of human diseases in a head-to-toe order was made by the Byzantine (Eastern Roman Empire) Greek physicians Oribasius of Pergamum (Ορειβάσιος ὁ Περγαμηνός, 325–403 AC), in his great encyclopedia “Ιατρικαί Συναγωγαί” (Collectiones medicae), composed by seventy volumes [15], and later by Paul of Aegina (Παύλος ὁ Αιγινήτης, 625–690 AC), in his medical compendium composed by seven books, called “Πραγματείας ιατρικής βιβλία επτά”, in Latin “Epitomae medicae libri septem” (On medicine, seven books), inspired on the Hippocratic and Oribasius thought. In these seven books, he classified all the known diseases, such as gastrointestinal illness, diseases related to human nutrition, fever, urogenital infectious diseases, parasitic infestations, but without any separation between external and internal diseases. He described also the therapies used at that time and the first surgical techniques [16]. According to him, “*… cancer was a kind of sensitive and painful swelling that has a black color, an ugly and irregular appearance and could be ulcerative. In addition, the cancerous tissue has vessels stretched in different directions. When forming in an organ that could be cut, cancer should be eradicated, and its scar cauterized* …” Later, during the 10th century aC, the Byzantine Greek physician Chrysobalantes Theophanes or Nonnos (*Χρυσοβαλάντης Θεοφάνης ή* *Νoννός*) wrote the “*Σύνοψις εν Επιτομή της ιατρικής απάσης τέχνης”* (Synopsis in an Epitome of all Medical Art), composed by 297 chapters with a large number of references to the previous physicians, such as Oribasius of Pergamum and others. He wrote about many diseases of his time and their possible therapies. Finally, the 13th century is characterized by the work of Nikolaus Mirepsus (*Νικόλαος Μυρεψός*) Actuarius *(Ακτουάριος*) and he was at the court of the *Emperor John III Doukas Vatatzes* (*Ιωάννη*ς *Γ’ Δούκα*ς Βατάτζης, 1222-1254 AC, *East Roman Empire*) which was great importance of the following years. He wrote a collection of prescriptions, the “*Μέγα Δυναμερόν*” (Great Dynameron or Medicamentorum Opus) which was the official text on drug therapy of his time and for the subsequent medical schools and one of the main sources of modern pharmacology. The collection classifies in twenty-four units, because the pharmaceutical recipes based always on the letters of the Hellenic Ionic alphabet (Α-Ω), from which the title of the recipe begins and it was translated into Latin and printed in the 16th century, and until the 17th century. In this collection text are described over two thousand various recipes such as those with psychoactive herbs, such as mandrake, or those with opium as a pain reliever, antitussive, and against hysteria (even in our day it is added to antitussive and analgesic formulations) and others for all types of diseases such as gastrointestinal ones.

Thus, although the head-to-toe classification was a feature of later Greek medicine, it was the Arabic medicine who developed it a lot and clearly separate external disease from internal ones. In the 11th century, a possible description of a gastric cancer is found in Avicenna’s (980-1037 AC) Medical Encyclopedia.

Ibn Sina (Persian: ابنسینا), also known as Abu Ali Sina (ابوعلیسینا), Pur Sina (پورسینا) and, in the west, known as Avicenna, was one of the most significant Persian physicians and the father of the modern Arabic medicine. His encyclopedia included all the medical knowledge of the time from both Greek and Islamic civilizations [17,18,19].

At the same time, we must mention the important medical text “Liber Pantechne” (παντεχνη, all medical arts), written by the Benedictine monk Constantine Africanus in the 11th century AC, scholar of the famous Schola Medica Salernitana (the most important medical school of the antiquity, based in Salerno, southern Italy, from the 9th to the 19th century), which is based on the works of many others physicians, such as Ibn Al-Jazzar (أبوجعفرأحمدبنأبيخالدبنالجزارالقيرواني, 898–980 AC), author of “*De stomacho*”.

The medieval medical knowledge radically changed with the Renaissance, probably because new scientific methods to study pathologies were allowed, such as autopsy. It was Morgagni, in 1761, who first used the dissection to link anatomo-pathological findings with the patient’s illness. He was an Italian anatomist who introduced the anatomo-clinical concept in medicine and established anatomy as the instrument to identify the seat and etiology of any disease. Thanks to this new scientific way to study cancer, the oncology field started to develop quickly [20]. In the 18th century, Dr. Peyrile published at the Academy of Lyon his thesis about the cancer origin, entitled “Dissertatio Academica de Cancro,” (1774); it has been considered the starting-point of the modern oncological era [8]. It was the Italian Lazzaro Spallanzani (1729–1799), in the 18th century, who first tried to explain the action of the gastric juice on foodstuffs [21].

The lack of knowledge was considered a possible explanation of the sudden and mysterious death of Napoleon Bonaparte in 1821. He began to suffer from recurrent episodes of fever, abdominal pain, vomiting, and persistent hiccupping two years before his death, when he vomited coffee-ground material, with progressive weakness, profuse sweating, sever hiccupping and tachycardia. In a lucid moment of his delirium, he asked his personal physician, Dr. Francesco Antonmarchi, anatomist and pathologist of Pisa University, to make an autopsy on his cadaver after his death and to examine his stomach, because gastric pathologies had caused the death of at least four of his relatives: grandfather, father, one sister, and one brother. He died few hours later for a massive bowel movement followed by a circulatory collapse. In the autopsy report, written by Dr. Antonmarchi, was reported a detailed description of what he saw “*... the volume of the stomach was small, its anterior surface seems to be normal but on the right side exists a close adhesion with the inferior face of the left liver. Near the small curvature there was a hard area, perforated in the center. The perforation was closed by the liver adhesion. On opening the organ along its large curvature its capacity appeared filled with a considerable quantity of matters mixed with a liquid resembling the sediment of coffee. The internal surface of the stomach was occupied by a cancerous ulcer whose center was on the lesser curve and the digitations were extended from the cardias ‘till 1 or 2 cm before the pylorus, with a scirrhous thickening of the wall* …” Previously, Napoleon had suffered vague abdominal symptoms, perhaps due to chronic gastritis which preceded his familial gastric cancer [8,22,23,24]. It is very curious that in many paintings representing the Emperor, he is portrayed with his hand on his abdomen, maybe for abdominal pain.

Other famous people lost their lives to gastric cancer, such as Marshal Joseph Pilsudski, the Polish hero of the World War I, who died for this cancer in 1935; the Irish writer James Joyce, who died of a gastric cancer in 1941; in Japan, China, Korea, and the other countries of the Pacific area, gastric cancer has been well known as one of the most important killers: it is considered endemic. To cite some examples, many members of the shogun Tokugawa family, who dominated the country between the 17th and the 19th centuries, were affected by this cancer; the founder of the dynasty, the first shogun Ieyasu Tokugawa, who unified Japan in 1615, probably died from gastric cancer in 1616. Died of this cancer also important personalities of the history such as King Charles the 11th of Sweden and Pope John XXIII [8].

During the Napoleon’s period, however, gastric cancers were formally unknown. After a decade from the Emperor’s death, benign and malignant gastric ulcers were described in 1835 in the texts of J. Cruveilhier, a French anatomist; in 1839, Robert Bayle made a detailed description of the pathology of gastric cancer in a treatise [25]. Between 1649 and 1652, two dutch physicians, Nicholas Tulp (1593–1674) and Zacutus Lusitani (1575–1642) affirmed that cancer was contagious because they observed many cases of cancer in members of the same household. They proposed to isolate cancer patients, preferably outside of cities, in order to prevent the spread of cancer. For this reason, Jean Godinot (1661–1739), canon of the cathedral in the French city of Rheims, accepted, in 1740, to transform his cathedral in the first cancer hospital for the poor for a considerable amount of money. It was called “*Hôpital des cancers*”; however, after few years, in 1779, the hospital was dislocated outside city to avoid multiples infections [26].

From the late 19th to the early 20th century, the physicians considered gastric cancer a consequence of an abdominal traumatic injury, so they started to cause mechanical trauma on experimental animals, in order to induce cancer in their stomachs. This theory was soon abandoned after many unsuccessful attempts.

The 19th century was characterized by many discoveries in the medical fields about gastrointestinal pathologies: in 1805, Philipp Bozzini was the first to observe inside the urinary tract, the rectum and the pharynx using a light-guiding instrument. He is considered the father of endoscopy, but it was only in 1868 that a German physician, Adolf Kussmaul, could watch inside the stomach with a gastroscope, and in 1883, Hugo Kronecker and Samuel Meltzer developed an esophageal manometer. Charles Emile Troisier (1844–1919) first described the enlarged left supraclavicular lymph node and its connection not only with gastric carcinoma but also with several other abdominal malignancies [27,28,29]. In 1823, William Prout discovered that stomach juices contain hydrochloric acid, which can be separated from gastric juice by distillation [30,31].

## 3. Gastric Surgery and the Development of Endoscopy

The official history of gastric cancer surgery began 40 years later, when the French surgeon Jules Pean attempted the first gastric resection in 1879; the patient, unfortunately, died few days after. In 1880, Ludwig von Rydygier, professor of surgery at Krakow University, tried another gastric resection, but it was unsuccessful. The first successful gastric resection was attempted by Theodor Billroth, one year later, in the Allgemeine Krankenhaus Clinic in Wien. The patient was a 43-old woman who survived four postoperative months. The operatory technique used by Billroth maintained his name still today as Billroth I, which consists in a gastro-duodenal anastomosis directly connecting the gastric stump to the duodenal one. This intervention, technically more difficult, consists in reconstructing the alimentary tract similarly to the physiological one. Billroth, soon after, modified this operation for a more radical resection, known as Billroth II. These types of resections are currently used in the modern gastric surgery, but not for oncologic purposes [32].

In 1897, the Swiss surgeon Karl Schlatter performed the first total gastrectomy for a diffuse gastric cancer in Zurich, at the surgical department directed by Kronlein. He removed the whole stomach and made an esophagojejunostomy for reconstruction. The operation was successful, and she died for recurrent tumor after 14 months. Total gastrectomy was then practiced all over the world with different techniques, first by Charles B. Brigham in San Francisco and by Richardson in Boston [8].

The continuous development of research and new discoveries in the field of gastroenterology and gastric surgery continued in the 20th century.

During the last century many hypotheses have been proposed to explain the etiology and pathophysiology of gastric cancer. Based on the current knowledges of the time, environmental, and dietary causes were proposed, as well as the increased risk in persons having group A blood or menopausal women, etc. Such etiopathogenetic links were variously explained but, in most cases, never confirmed differing from the *Helicobacter pylori* infection that is currently confirmed as the main cause of gastric diseases, including cancer. Table 1 summarizes the main causes of gastric cancer proposed during the time.

In 1915 a small amount of gastric juice was taken from a human stomach to study its characteristics by Jesse McClendon. Gastrointestinal endoscopy has passed through three principal changes in the forms of endoscopes used to examine the gastrointestinal tract: the “rigid endoscope” era (1805–1932); the “semi-flexible endoscope” or “Schindler” era (1932–1957); and the “fiber-optic” era (1957–present) [33].

Many important steps forward and progresses were made by scientists in the field of human nutrition and digestive system, researching about the chemical processes and the biochemical mechanism of physiological digestion, but also about the unknown etiopathogenetic agents that could cause the stomach cancer. In particular, based on previous demonstrations of close relationships of bacteria with cancers and tumor-like conditions, many pathologists and microbiologists were aiming to find one or more microorganisms related to gastric cancer [34,35,36].

Before the 1950s, there were many microbiological descriptions of bacteria in the stomach and in gastric acid secretions, lending credence to both the infective theory and the hyperacidity theory as being causes of peptic ulcer disease: from the theory of professor Francois de la Boe Sylvius, a Flemish scientist born in Hanau (currently a German city), who believed that acidic lymph fluid was the cause of cancers, to the theory of his contemporary, Nicolaes Tulp, who believed that cancer was a contagious poison that slowly spreads in the whole body. It was just in 1926 that Johanes Andreas Fibiger (1867–1928), professor of pathology in University of Copenhagen, received the Nobel Prize for discovering the etiopathogenetic agent causing the stomach cancer. He demonstrated that the roundworm which he called “*Spiroptera carcinoma*” could cause stomach cancer in rats and mice. He supposed this was the cause of gastric cancer because he observed inflammatory and degenerative mucosal changes in the rats’ stomach after feeding them with infected cockroaches. German scientists, such as Walter Krienitz, found spiral-shaped bacteria in the lining of the human stomach in 1875, but they were unable to culture them, and the results were eventually forgotten [35].

In 1915, Rosenow published his theory after he infected a large number of experiments animals with *streptococci*. In his paper [36], he demonstrated *streptococcus* as an etiologic factor in nine diseases, including gastric and duodenal ulcer, due to its affinity for the gastric mucosa.

## 4. The Discovery of the *Helicobacter pylori* and Its Relationship with Gastric Cancer

It was a significant turning point for the history of the gastric cancer when Barry J. Marshall and Robin Warren, two Australian researchers, discovered the bacterium *Helicobacter pylori* and deciphered its role in gastritis, peptic ulcer disease (PUD) and gastric tumors. For their great work they were awarded in 2005 the Nobel Prize in Physiology or Medicine [37]. Their studies revealed that gastritis, gastric and duodenal ulcerations and some type of gastric cancers were the result of infection with some curved, Gram-negative bacilli. The Gram negative curved bacillus *H. pylori* has become the prize bug of all times. Barry Marshall and Robin Warren the two discoverers of this organism have been awarded with this year’s Nobel Prize. The Nobel committee at the Karolinska Institute of Sweden has selected this paradigm shift discovery of 1982 as the most impacting in medical sciences. This award has surprised many as the Nobel assembly has selected this ‘Robert Koch styled medical detective work’ for the prize as compared to many outstanding basic research stories on the waitlist. This editorial briefly touches the significant impact of *H. pylori* on gastroduodenal management and the path forward as the bug has become quite controversial in recent times. Bacteria colonizes the human stomach and periodontal pockets, causing a chronic active gastritis, which can complicate with peptic ulcer disease [38]. Before they announced their findings, a large number of physicians thought that stress and lifestyle factors were the major causes of these diseases. Warren and Marshall, at the first time, were regarded with skepticism and a lot of criticism but their major breakthroughs were soon widely accepted. To enforce their theory about the linkage between the *Helicobacter* and gastro-duodenal pathologies, Warren infected himself with the bacterium and developed the classical symptoms. This ‘self-help’ experiment was published in the Medical Journal of Australia [39]. In the 1994 the World Health Organization (WHO) and the International Agency for Research on Cancer (IARC) declared *H. pylori* a Group 1 carcinogen.

A Greek physician, Jonh Lykoudis (Ιωάννης Λυκούδης, 1910–1980), who was himself affected by gastritis and peptic ulcer, in 1968 developed a treatment which consisted of three types of antibiotics (2 quinolines, 5,7-diiodo-8-oxyquinoleine 0.125g; 5-iodo-7-chloro-8-oxyquinoleine 0.125g, and streptomycin sulfate 0.075g), with vitamin A 10,000 UI. He named these pills “*Elgaco*” (from the Greek term ἕλκος = ulcer, and the two words γαστρίτιδα = gastritis and κωλίτης = colitis). He used these pills as a therapy for gastritis and gastric ulcer, 6–8 times daily for 10 days, because he was convinced that the real cause of these diseases was infectious, but without knowing specifically what kind of infectious agent was involved. Vitamin A was included to increase the mucosal regeneration. He started treating thousands of patients (around 30,000) with great results and no toxicity. Despite this treatment being a successful one, he did not make a real clinical trial, so each treatment and outcome was not scientifically resumed. In 1966, Lykoudis attempted to publish his observations “Ulcer of Stomach and Duodenum” in the Journal of the American Medical Association, but it was rejected. Unfortunately, no copy of this text survived [40].

The actual “*Helicobacter pylori*” was originally named “*Campylobacter pyloridis*” and then “*Campylobacter pylori*” in 1987 (pylori is the circular opening leading from the stomach into the duodenum, from the Greek word “πυλωρός” = gatekeeper) [41,42]. His RNA analysis showed, in 1989, that the bacterium did not belong in the genus *Campylobacter*, so it was placed in the genus *Helicobacter* (from the Greek word *έλιξ* = spiral and *βακτήριον* = bacterium) [43,44].

In 1987, Thomas Borody, an Australian physician, proposed the first triple therapy for the treatment of duodenal ulcers [45]. In 1994, the National Institute of Health recommended antibiotics in the treatment of recurrent duodenal and gastric ulcers caused by *H. pylori*. Peptic ulcer disease associated with *Helicobacter* is currently treated with antibiotics to eradicate the infection and to allow the ulcer to heal. Previously, the only option was symptom control using antacids, H2-antagonists or proton pump inhibitors alone. Nowadays, he first-line therapy is the “triple therapy”, based on the use of a combination of a proton-pump inhibitor and two antibiotics, clarithromycin and amoxicillin. In people who are allergic to penicillin, amoxicillin can be replaced with metronidazole. Other options are available in case of treatment failure for antibiotic-resistant bacteria: a “quadruple therapy”, which adds a proton pump inhibitor, two antibiotics and a bismuth colloid, such as bismuth subsalicylate. In case of treatment failure for antibiotics resistance, which is an increasing problem in *H. pylori* infections, they can be replaced by the use of another antibiotic to which the bacteria is sensitive; for the treatment of clarithromycin-resistance, the use of levofloxacin has been suggested [46,47]. It has been demonstrated that gastric mucosa-associated tissue lymphoma (MALT lymphoma, or MALToma), a rare mature B-cell neoplasm is associated with *H. pylori* infection. It is one of the rare and incredible cases of tumors curable by antibiotics therapy alone [48,49,50,51].

Table 2 summarizes the evolution of medical knowledge about gastric conditions from ancient Egypt to the discovery of *Helicobacter pylori* and its possible cure.

## 5. Towards the Future

Omics has changed our understanding of the biology of many diseases. The progress of knowledge in this field is very rapid, either in terms of new basic scientific knowledges or their clinical application. One of the most important knowledge of molecular biology of the last decade is the different and ubiquitous role of small RNA molecules in gene regulation. Many studies suggest that this class of molecules contributes to the pathogenesis of diseases, especially in cancer and immunological disorders, and tests targeted to the identification of patterns of miRNA expression in gastric cancers might be added to traditional techniques in the next future to determine their origin and to ameliorate prognosis and therapy.

## 6. Conclusions

Gastric cancer is probably a very ancient disease, that has not been recognized by the past’s civilizations for the lack of histological and pathophysiologic knowledge. It was considered a divine punishment from Egyptian culture, as something who attacked the body from the outside by Greek physicians. From the Renaissance, a large number of scientific discoveries all over the world brought actual knowledge about all the pathophysiologic mechanisms which can cause gastric mucosal alterations and the development of the duodenal and gastric ulcers. Many efforts have been made to investigate about the risk factors of gastric cancer and to the surgical techniques to obtain an even more radical result, especially in those countries where this cancer is one of the first leading cause of cancer death, such as Japan. However, the role of the resident microbiota in the regulation of the immune system and oncogenesis, especially gut microbiota, is continuously growing [52,53,54].

Special attention has been dedicated to the therapies for this condition and their consequences. In fact, due to the elimination of an extensive part or of the whole stomach, many nutrients became no more available, worsening the clinical condition immediately after, as well as for lifespan. Possible consequences include post-surgical sepsis, malabsorption, dumping syndrome, etc. [55,56,57,58,59].

Based on the well-known positive effects as supportive therapy in a large number of infective and inflammatory conditions, new horizons in research are focused to investigate the potential role of the lactic acid bacteria ingestion for their suppressive effect on *H. pylori* infection in both animals and humans [60,61,62,63,64,65].

In fact, it has been demonstrated that eating yogurt supplemented with *Lactobacillus* and *Bifidobacterium* improves the rates of eradication of *H. pylori* in humans, confirming and expanding our knowledge about the role of our microbiota in the maintenance of health [66,67,68]. This is due to the probiotics production of beneficial substances for the gastrointestinal system, such as butyrate, which help suppress *H. pylori* infections as an adjunct to antibiotic therapy. The availability of molecular diagnostic tools and continuous scientific progresses allow us to test and detect rapidly the possible presence of many microorganisms in any tissue.

In fact, the cytokine- and cell-adhesion-dependent bone marrow niche and stromal microenvironment support new vessel formation and cancer proliferation, irrespective of immune-surveillance [69].

Recently, some medical researchers are investigating about the possible role of others infective agents, such as the Human Papilloma Virus (HPV) and the Epstein–Barr Virus (EBV), when they infected human cells together with *H. pylori* [70,71,72].

For instance, EBV appears to impact the immune-environment and the patient’s survival [73], is implicated in the immune status equilibrium, a major driver of gastric cancer (GC) initiation [74]. This intimate interaction between GC cell, microenvironment via bystanders (i.e., endothelial cells), and CD8+ T cells creates a permissive immune microenvironment that allows undisturbed cancer proliferation in both solid and hematological malignancies [75,76].

The prevailing point of view has been that N-terminally truncated p53 family isoforms (ΔNp53, ΔNp63, and DNp73) predominantly counteract cell cycle arrest and apoptosis. Recent progress in the field extend these well-known functions and place these isoforms in the center of a comprehensive regulatory network controlling major epithelial-to-mesenchymal transition (EMT)-relevant signaling pathways [such as transforming growth factor-β (TGF-β), wingless-int (WNT), insulin-like growth factor (IGF), and signal transducer and activator of transcription (STAT)), microRNAs, and EMT-associated transcription factors that promote invasion, loss of tumor cell polarity, and metastatic behavior in conjunction with a chemo-resistant phenotype [77].

In frame of this thinking, one open scientific debate is ongoing about the translational value of immune microenvironment in GC. Indeed, immune microenvironment, antigen stimuli and antigen-presenting cells, such as dendritic cells appear to be connected in a complex network involving WNT/CTNNB1 pathway, a well-known driver of cancer aggressiveness and tumor-dissemination with fundamental implication for immune landscape, therapeutic approaches and patient molecular stratification in a plethora of solid tumors [78,79].

These observations add new weight to the concept that currently underappreciated truncated forms of this tumor suppressor family play an equally important role in promoting cancer aggressiveness as do mutant p53 proteins, also exemplifying how the consequences of ΔN/DN expression depend on cellular contexts [76].

Many efforts must be made in the future to contrast this terrible human disease, which still kills thousands of victims every year. New molecular techniques and new knowledge about the causes of gastric carcinoma, as well as about its microenvironment will help researchers to understand how to fight and, moreover, prevent this killer at best [80]. As a practical consequence, a continuous and synergic cooperation between health professionals from many medical specialty fields (gastroenterologists, surgeons, immune-oncologists, periodontists, pharmacologists, etc.) will be needed for the management of a personalized approach to patients with gastric cancer.

## Figures and Tables

**Table 1:** Summary of proposed causes of gastric cancer. During the 20th century gastric cancer was considered a consequence of preexisting or coexisting conditions (i.e., chronic atrophic gastritis), poor lifestyles, or paraphysiological conditions (i.e., diet poor in fibers, menopause, etc.), and to a genetic predisposition (gene mutations, group A blood, etc.). Most of these links were never confirmed, except the causative role of *H. pylori*.

Precursor Conditions	Enviromental Factors	Genetic Factors
H. Pylori infection (CagA positive strains) Pernicious anemia Gastroesophageal reflux Barrett’s esophagus Chronic atrophic gastritis and intestinal metaplasia Ménétrier disease Gastric adenomatous polyps Previous gastric surgery (especially in partial gastrectomy: high risk of cancer of the gastric stump)	Lifestyle (alcohol, tobacco smokers, obesity, low physical activity) Low socioeconomic status Dietary factors (diets rich in salt/sodium, rich in starch and poor in protein, smoked or poor preserved foods, low intake of fruit and vegetables) Occupational exposures (workers processing rubber, asbestos and timber, or farming, mining, refining, as well as exposure to dusty and high temperature environments as in wood processing plant operators, cooks, food and related products machine) Estrogens decrease with menopause Viral infections (EBV, HBV?) Radiations	Family history of stomach cancer Elderly (for degenerative changes and accumulated DNA damages) Hereditary non-polyposis colon cancer Li-Fraumeni syndrome Downregulation of E-cadherin expression Interleukin-1B gene mutation Hypo-gamma-globulinemia (primary immunodeficiency) Group A blood

**Table 2:** Overview of the main steps of the knowledge about gastric cancer and its therapy.

2125 BC	First researches in gastoenterological field during the 10th dynasty of the Pharaos by Irynathty, the court physician
1600 BC	First descriptions of cancer in the “Kahun (or Fayoum)”, “Edwin Smith” and “Ebers” papyri
1200 BC	Earliest complete skeleton with multiple osteolytic metastatic bone lesions found in northern Sudan
6th century BC	Alcmaeon of Croton’s studies on gastrointestinal mechanisms and digestive functions through animal’s vivisection
4th century BC	First use of terms “καρκίνος” (karkìnos=cancer) and “καρκίνωμα” (karkìnoma= carcinoma) by Hippocrates
3rd century BC	Development of human anatomy’s knowledge through dissections on cadavers made by Herophilus of Chalcedon and Erisistratus of Ceos
1st century BC–1st century AC	Use of drinks based on plant’s extracts for gastrointestinal symptoms by Asclepiades and Dioscorides
2nd century AC	Introduction by Galen of the prefix “onco-” (*όγκο*-) and the suffix “-oma” (*-ομα*) to describe tumors in general, reserving the term “carcinoma” for the malignant ones
3rd–6th century AC	Classification of gastrointestinal diseases in the Oribasius Encyclopedia and in the Paul of Aegina’s Compendium
11th century AC	Avicenna’s Encyclopedia included all the Arabic medical knowledge of the time
15–17th century AC	“*Hôpital des cancers*” were created in France to isolate cancerous patients, considering cancer a contagious disease
17th century AC	Autopsy was allowed as a legal method for medical studies
18th century AC	Dr. Peyrile published his thesis about the cancer’s origins, starting-point of the modern oncological era
1805	Rigid endoscope was first used to explore the gastrointestinal tract
1881	First successful gastric resection was attempted by Theodor Billroth
1897	First successful total gastrectomy was attempted by Karl Schlatter for a diffuse gastric cancer in Zurich
1926	J.A. Fibiger received the Nobel Prize for demonstrating the “Spiroptera carcinoma”
1932–1957	Semi-flexible endoscope first and then fiber-optic one were first used to explore the gastrointestinal tract
1968	J. Lykoudis developed a treatment (Elgaco) based on antibiotics and vitamin A to treat peptic ulcers
1982	B.J.Marshall and R.Warren recognized Helicobacter pylori as the cause of gastritis and peptic ulcers
1987	T.Bodory proposed a triple therapy for the treatment of duodenal ulcers
1994	WHO and IARC declared H.pylori a Group 1 carcinogen. NIH recommended antibiotics for the treatment of recurrent duodenal and gastric ulcers caused by this bacterium.
2005	B.J.Marshall and R.Warren received the Nobel Prize in Physiology or Medicine
